# New Approaches and Biomarker Candidates for the Early Detection of Ovarian Cancer

**DOI:** 10.3389/fbioe.2022.819183

**Published:** 2022-02-10

**Authors:** K. R. Hossain, J. D. Escobar Bermeo, K. Warton, S. M. Valenzuela

**Affiliations:** ^1^ School of Life Sciences, Faculty of Science, University of Technology Sydney, Sydney, NSW, Australia; ^2^ ARC Research Hub for Integrated Device for End-user Analysis at Low-levels (IDEAL), Faculty of Science, University of Technology Sydney, Sydney, NSW, Australia; ^3^ School of Women’s and Children’s Health, Faculty of Medicine and Health, University of New South Wales, South Wales, NSW, Australia

**Keywords:** ovarian cancer, CLIC proteins, exosomes, diagnostics, biomarkers

## Ovarian Cancer

Ovarian cancer (OC) is a disease that most often affects post-menopausal women who present abdominal discomfort and bloating over a few months prior to detection. The majority of patients are diagnosed at advanced stages of the disease as the early stages are commonly asymptomatic (Jayson et al., 2014). According to Global Cancer Statistics 2020, OC is the seventh most common cancer in women worldwide accounting for around 314,000 new cases (3.4% of all new cancer cases in females) annually (Sung et al., 2021). In 2021 it established itself as the ninth most commonly diagnosed cancer amongst Australian women with 1720 new cases and 1,042 reported deaths (www.canceraustralia.gov.au). Prognosis is significantly determined by the stage of diagnosis where survival for stages I, II, III and IV is 73–92%, 45–55%, 21% and <6%, respectively^1^, underscoring the need for better early detection of ovarian cancer.

There are four main types of OC: epithelial, Germ cell, sex chord and stromal, with each having different epidemiological statistics, and with epithelial ovarian cancer (EOC) accounting for approximately 90% of all cases (Smith et al., 2006). In EOC there are histological subtypes, though most patients have high-grade serous ovarian cancer, a disease characterised by p53 gene abnormalities (Köbel, 2010). Some high grade serous ovarian cancers are caused by deleterious mutations of BRCA1 and BRCA2 genes, while others arise from a combination of somatic mutations (Network, 2011). The most common and effective management of the disease is a combination of surgery with chemotherapy. Surgical removal of cancer mass almost always occurs after diagnosis however, this is not always feasible when the cancer is very advanced. Furthermore, although surgery has proven effective in early stages of the disease, most patients diagnosed at advanced stages will go on to develop many iterations of recurrent disease (Jayson et al., 2014).

Cancer Antigen-125 (CA-125) and trans-vaginal imaging are currently routinely used as part of ovarian cancer diagnosis. Blood level of CA-125 is the most widely used serum biomarker, but it lacks the sensitivity or specificity to be used alone as a screening test (Jayson et al., 2014). It is also not useful for early diagnosis as CA-125 expression levels are too low for accurate detection and there are also several other conditions including endometriosis (Nisenblat et al., 2016), gall bladder (Wang, 2014) and liver cancer (Devarbhavi et al., 2002) where CA-125 levels are elevated, leading to false positive results. Likewise, it is often difficult to detect small early-stage tumors with trans-vaginal imaging. Hence, diagnosis often involves invasive techniques like laporascopy and tissue biopsies. It is therefore clear that early diagnosis of ovarian cancer requires improved screening tests that can be performed easily and inexpensively, as well as achieving high sensitivity and specificity.

## New Advances in the Screening of Ovarian Cancer

Several different approaches have been proposed to combat the shortcomings of current techniques. Studies focused on smaller molecules, such as circulating miRNA and cell-free DNA (cf-DNA) have gained significant attention in recent times (Jørgensen, 2013; Ling et al., 2013; Peng and Croce, 2016). These molecules have emerged as promising cancer biomarkers suited for improved non/minimally invasive diagnostic, prognostic and therapeutic applications. Multiple miRNAs associated with ovarian cancer, such as the Let-7 family miRNAs, together with a variety of miRNA assays including RT-qPCR, microarrays and RNA sequencing have been reported as potential screening techniques (Banno et al., 2014; Deb et al., 2018; Aziz et al., 2020). As of 2021 there were over 250 miRNAs associated with ovarian cancers on the online database miRCancer, and depending on the cellular context, their up or down regulation was shown to be involved in disease progression (Xie et al., 2013). Despite demonstrating good analytical performance, a lack of reproducibility and the high costs associated with miRNA profiling mean their development remains a work in progress [for in-depth discussion of these challenges, see reviews (Tiberio et al., 2015; Witwer, 2015)]. In response to these issues, the research focus has expanded to include alternate approaches like the use of exosomes (EX) which are gaining increased interest in recent literature.

EXs are extracellular vesicles, ranging between 40 and 160 nm in size (Ruivo et al., 2017), released from the phospholipid membrane of the cell. These lipid bilayer-bound vehicles carry nucleic acids, proteins, and lipids to neighbouring or distant cells that influence a multitude of processes (Schey et al., 2015). Since EXs mimic the profile of the host cell from which they originate, it is not surprising that EXs released from cancer cells have been shown to aid in tumor progression including cellular proliferation, angiogenesis, migration, invasion, metastasis, and drug resistance (Tai et al., 2018). These properties make them suitable targets not only for novel therapeutics but also as specific markers for use in cancer diagnostics, prognosis, and even as chemotherapy drug-delivery systems. A recent review by Huda et al. (2021) highlighted several clinical applications and preclinical advancement outlining the importance of exosomes for use in targeted drug delivery, biomarker study, and vaccine development (Huda et al., 2021). A recent study by Zhao et al. (2016) reported the development of a microfluidic chip to isolate EXs expressing certain ovarian cancer biomarkers including CA-125 (Zhao et al., 2016). In addition, use of specific antibodies resulted in a greater number of exosomes being captured in patient samples, compared to healthy controls (Zhao et al., 2016). Although this study highlighted how exosomal CA-125 may be used to distinguish between healthy controls and cancer patients (Zhao et al., 2016), the associated drawbacks of CA-125 as a promising ovarian cancer biomarker for early detection remain a challenge.

It is now well established that the proteomic profile of EXs can act as a useful tool or biomarker for diagnosing various types of cancer (Nuzhat et al., 2017; Sharma et al., 2017; Kok and Yu, 2020; Croft et al., 2021). For example, a study by Klein-Scory et al. (2014) illustrated that EXs secreted from pancreatic cancer cells had a distinctive proteomic profile allowing for possible use as an early stage biomarker (Klein-Scory et al., 2014). Thus, it is seemingly reasonable to exploit the proteomic profile of exosomes from ovarian cancer cells to identify novel biomarkers. One such example is the Chloride Intracellular Ion Channels (CLIC) family of proteins which has been shown to be elevated in patients with ovarian cancer (Kobayashi et al., 2012).

## Chloride Intracellular Ion Channels as Biomarkers for Ovarian Cancer

The CLIC family in vertebrates consists of six evolutionarily conserved protein members (Littler et al., 2010). These proteins are unusual, existing in cells as both soluble and membrane bound proteins, where they demonstrate “moonlighting” activity, exhibiting two independent functions. In the membrane bound form, CLIC proteins act as ion channels (Valenzuela et al., 2013) while in the soluble form, they act as oxidoreductase enzymes, which are most likely involved in cell protective functions like detoxification and/or anti-oxidative roles (Al Khamici et al., 2015). Several studies have also shown CLIC proteins to have important extracellular activities (see review (Argenzio and Moolenaar, 2016)), with CLIC1, CLIC3 and CLIC4 found on endometrium/placenta cell exosomes (Zhao et al., 2016). CLICs are also known to be up-regulated in various types of cancers where they are primarily involved in cancer metastasis *via* exosomal and anti-apoptotic activities (GururajaRao et al., 2020). In a recent bioinformatics study of patient survival as a function of the relative mRNA levels, across several human cancers, the data clearly indicates both low or high CLIC expression levels, influence patient survival (GururajaRao et al., 2020). For example, high CLIC1 and CLIC3 mRNA expression were associated with advanced stages of liver cancer (Huang et al., 2021) while high CLIC4 mRNA expression correlates with poor patient survival in EOC (Singha et al., 2018). However, to better understand the therapeutic potential of these proteins, it is important to further deduce details regarding their tissue expression and release into blood.

Studies have shown two members of the CLIC protein family, CLIC1 and CLIC4, to be significantly upregulated in EOC patients compared to healthy controls (Tang et al., 2012; Tang et al., 2013; Ye et al., 2015; Singha et al., 2018; Yu et al., 2018), and shed into the blood of ovarian cancer patients (Tang et al., 2012; Tang et al., 2013). At the tissue level, combining CLIC1 and CLIC4 staining with CA-125, resulted in better sensitivity, compared to using CA-125 alone, hence a panel consisting of CLIC proteins and CA-125 may outperform individual biomarkers (Singha et al., 2018). Expression levels of CLIC1 and CLIC4 have also been implicated in patient survival, with elevated CLIC4 expression a negative indicator of patient survival in ovarian cancer (Singha et al., 2018). Knockdown of either CLIC4 or CLIC1 resulted in slower growth of ovarian cancer cells suggesting a role of CLICs in cell proliferation and cell migration (Singha et al., 2018). Furthermore, consistent with other reports, these proteins have also been shown to play important roles in EOC progression across multiple EOC subtypes (Ye et al., 2015; Singha et al., 2018; Yu et al., 2018). Similarly, CLIC3 levels have been shown to be elevated in 90% of ovarian cancer patients, where upregulation of CLIC3 in breast cancer tissue was associated with increased cancer cell invasiveness (Hernandez-Fernaud et al., 2017). The fact that exosome associated CLICs display cancer-specific signatures for EOCs, allows for the coupling of CLIC expression with emerging technologies capable of detecting and profiling exosomes, leading to simple, non-invasive, early and specific detection of ovarian cancer.

## Diagnostic Approaches for the Analysis of Exosome Associated CLICs as Biomarkers for Early Detection of Ovarian Cancer

**FIGURE 1:**
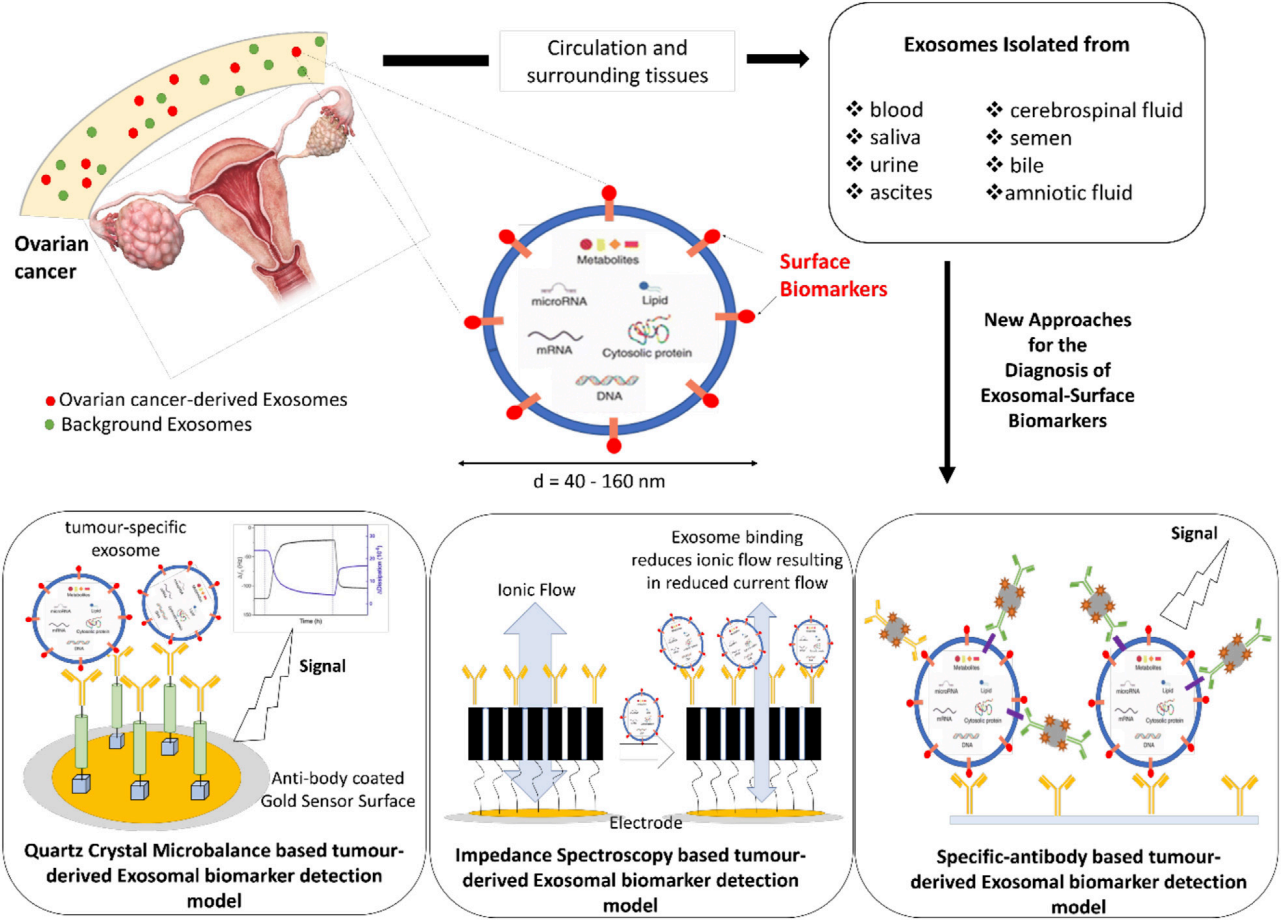
Schematic representation of new diagnostic approaches for the detection of Exosomal-Surface Biomarkers.

EXs have been successfully isolated from blood, urine, ascites, cerebrospinal fluid, amniotic fluid, semen, saliva, and bile (Colombo et al., 2014; Sullivan et al., 2017) thus providing an array of possibilities for their detection using several different techniques, with some examples as highlighted in Figure 1. Some of the currently available techniques that analyse EX proteins from different human body fluids, with or without EX isolation, include flow cytometry, protein microarray (EX Array), diagnostic magnetic resonance, nanoplasmonic sensing technology and microfluidics (Orozco and Lewis, 2010; Shao et al., 2012; Jørgensen, 2013; Im et al., 2014; Zhao et al., 2016; Sullivan et al., 2017). Recently, a number of studies have proposed exosomal proteins as diagnostic biomarkers of breast cancer (Rupp et al., 2011; Soung et al., 2017; Sullivan et al., 2017), where one such study showed EXs isolated using anti-CD24 and anti-EpCAM-coupled magnetic beads as potential breast cancer-specific markers (Rupp et al., 2011). An assay called “ExoScreen” available for the detection of colon cancer is based on the detection of cancer-specific circulating double-positive (CD146/CD9) EXs using photosensitive-beads, from as little as 5 µL of patient serum (Yoshioka et al., 2014). Numerous commercial kits are also available where specific EXs are isolated and analysed using microfluidic platforms for detecting different types of cancer (Contreras-Naranjo et al., 2017).

In the past several years, various electrochemical assays have been developed for the detection and characterisation of exosomes. One such development is an integrated magneto-electrochemical sensor (iMEX) where antibody-coated-magnetic beads are used for EXs capture and labelling following detection via electrochemical sensing (Jeong et al., 2016). Doldan et al. have developed an electrochemical sandwich approach using gold electrodes prefunctionalised with specific antibodies for exosome determination (Doldán, 2016). Electrical impedance technology is another well-established technique, which over the last 30 years has gained increasing interest in its applications in medicine. Impedance biosensors are usually label free and do not depend on any specific enzyme for analyte detection. Instead, impedimetric biosensors rely on unique bioreceptors or tags, which when specifically bound to the biomarker, produce changes in an electric current that lead to detection and in some cases, quantification of the biomarkers (Leva-Bueno et al., 2020). Impedance spectroscopy with tethered membrane technology has also been used for *E. coli* and *Staphylococcus aureus* detection, where specific antibodies embedded in the membrane detect the presence of bacteria in different fluid samples (Tan et al., 2011). This technology has been adapted to detect prostate (Mishra et al., 2012) and skin (Braun et al., 2017) cancer specific biomarkers and has the potential to be reliable, specific and selective, while being inexpensive and fast. Similarly, the detection of disease-related biomarkers based on acoustic sensors has created global research interest, with biosensors based on the quartz crystal microbalance (QCM) exploited for cancer diagnosis. Commercial QCM systems have been utilised for detecting highly metastatic human breast cancer cells (Atay et al., 2016; Bakhshpour et al., 2019), while an aptamer-based QCM was applied for the detection of leukemia cells (Shan et al., 2014). QCM technology with different signal enhancing approaches has been employed for the detection of several cancer biomarkers such as the carcinoembryonic antigen (CEA) for colorectal cancer (Chi et al., 2020), potential cancer biomarker poly (ADP-ribose) polymerase-1 (PARP-1) (Yang et al., 2019) and even for the detection of volatile compounds like propanol, ethyl benzene, hexanal and decane which are under investigation as non-invasive lung cancer biomarkers (AthirahAwatif Abdul Rahman et al., 2019). Recently, Suther et al. (2020) highlighted the potential of a QCM with dissipation monitoring (QCM-D) system to detect exosomal-CD63 biomarker by exploiting their surface protein profile (Suthar et al., 2020). Thus, the QCM system has emerged as a robust biosensing platform which allows the detection and quantification of a wide range of biomolecules. This, together with their high sensitivity and short detection time, makes QCM based-biosensors attractive for the early detection of various cancer types and the routine monitoring of disease progression.

Achieving early detection of ovarian cancer remains the greatest hope for improved patient survival outcomes. Therefore, investigation of these novel tools and technologies for early ovarian cancer diagnosis is a high priority.

## Footnotes

1 UK, C.R. Cancer Research UK, 2013.
